# Ecological application of biotic resistance to control the invasion of an invasive plant, *Ageratina altissima*

**DOI:** 10.1002/ece3.2799

**Published:** 2017-03-02

**Authors:** Chaeho Byun, Eun Ju Lee

**Affiliations:** ^1^ School of Civil and Environmental EngineeringYonsei UniversitySeoulKorea; ^2^ School of Biological SciencesSeoul National UniversitySeoulKorea

**Keywords:** *Ageratina altissima*, diversity–interaction model, *Eupatorium rugosum*, functional group, invasive plant management, priority effect

## Abstract

Biotic resistance is the ability of species in a community to limit the invasion of other species. However, biotic resistance is not widely used to control invasive plants. Experimental, functional, and modeling approaches were combined to investigate the processes of invasion by *Ageratina altissima* (white snakeroot)*,* a model invasive species in South Korea. We hypothesized that (1) functional group identity would be a good predictor of biotic resistance to *A. altissima*, whereas a species identity effect would be redundant within a functional group, and (2) mixtures of species would be more resistant to invasion than monocultures. We classified 37 species of native plants into three functional groups based on seven functional traits. The classification of functional groups was based primarily on differences in life longevity and woodiness. A competition experiment was conducted based on an additive competition design with *A. altissima* and monocultures or mixtures of resident plants. As an indicator of biotic resistance, we calculated a relative competition index (RCI_avg_) based on the average performance of *A. altissima* in a competition treatment compared with that of the control where only seeds of *A. altissima* were sown. To further explain the effect of diversity, we tested several diversity–interaction models. In monoculture treatments, RCI_avg_ of resident plants was significantly different among functional groups but not within each functional group. Fast‐growing annuals (FG1) had the highest RCI_avg_, suggesting priority effects (niche pre‐emption). RCI_avg_ of resident plants was significantly greater in a mixture than in a monoculture. According to the diversity–interaction models, species interaction patterns in mixtures were best described by interactions between functional groups, which implied niche partitioning. Functional group identity and diversity of resident plant communities were good indicators of biotic resistance to invasion by introduced *A. altissima*, with the underlying mechanisms likely niche pre‐emption and niche partitioning. This method has most potential in assisted restoration contexts, where there is a desire to reintroduce natives or boost their population size due to some previous level of degradation.

## 1 Introduction

Plant species are often transported outside of their native range, and some of these plants will naturalize without creating major problems (Lavoie, Saint‐Louis, Guay, Groeneveld, & Villeneuve, 2012; Thomas & Palmer, 2015). However, others are invasive species that can replace natives, alter habitat structure, and interfere with biogeochemical processes (Blossey, 1999; Mack et al., 2000). We often attempt to manage these species to minimize consequences to native species and ecosystems because invasive plants damage ecosystem functions and services (Castro‐Díez, Pauchard, Traveset, & Vil, 2016; Mack et al., 2000; Parker et al., 1999; Simberloff, 2005). Invasive plants also negatively affect the biodiversity of native communities (Lambert, Dudley, & Saltonstall, 2010; Matsuzaki, Sasaki, & Akasaka, 2016).

*Ageratina altissima*, white snakeroot (also known as *Eupatorium rugosum*), is an invasive plant in South Korea (Kil et al., 2004). White snakeroot is a perennial herb native to the eastern United States and Canada that is currently receiving much attention for its rapid invasion of Korean forests (Chun, Lee, & Lee, 2001; Lee, Han, Hong, & Choi, 2005). Populations of *A. altissima* are distributed in forest edges disturbed by the development of roads and human settlements from which the plant extends to inner forest patches, although individuals are scattered (Song, Hong, Kim, Byun, & Gin, 2005). Poisoning (milk sickness) in humans usually occurs following the consumption of milk or milk products from cows that consumed *A. altissima* (Davis et al., 2015 *)*.

Invasive plants, including *A. altissima*, are commonly controlled by mowing, burning, or applying herbicide (Derr, 2008; Kettenring & Adams, 2011). Controlling invasive plants requires repeated application of herbicide (Derr, 2008) or covering with black plastic for solarization (Marushia & Allen, 2011). Herbicide application is expensive and contributes to other environmental problems such as bioaccumulation in food web systems. After establishment, management costs for invasive species increase dramatically, and, when an invasive plant creates a dense mat of rhizomes and is ready to spread, complete eradication becomes almost impossible. Furthermore, eradication of an invasive plant does not guarantee natural recovery of native plants (Reid, Morin, Downey, French, & Virtue, 2009; but see also Thomsen, Brownell, Groshek, & Kirsch, 2012; Case, Harrison, & Cornell, 2016). Moreover, methods of eradication can create a disturbance on bare ground, which facilitates re‐invasion (Buckley, Bolker, & Rees, 2007; Iannone & Galatowitsch, 2008).

Therefore, management strategies should prioritize methods of prevention over those of eradication. Prevention is the most cost‐effective method. For example, sowing seeds of native species to reintroduce propagules can increase biotic resistance to invasion (Bakker & Wilson, 2004), and the evidence is increasing that sowing seeds of native species prevents or slows the invasion of invasive plants (Byun, De Blois, & Brisson, 2013, 2015; Kettenring & Adams, 2011; Middleton, Bever, & Schultz, 2010). Although no case study has examined the effect of biotic resistance on *A. altissima*, some studies examined the effects of restoration of native plants on the control of other invasive plant species, such as *Phragmites australis* (Byun et al., 2013, 2015; Peter & Burdick, 2010) and *Phalaris arundinacea* (Iannone & Galatowitsch, 2008; Perry, Galatowitsch, & Rosen, 2004; Reinhardt Adams & Galatowitsch, 2008).

Ecological theory provides an opportunity to develop restoration strategies based on how species assemble and regulate invasions (Funk, Cleland, Suding, & Zavaleta, 2008; Laughlin, 2014; Shea & Chesson, 2002; Zedler, 2005). A variety of theories and mechanisms are proposed, but two mechanisms are particularly relevant: competition‐based biotic resistance and diversity effect.

First, the mechanism of competition‐based biotic resistance is a function of which species are the most resistant to invasive plants. Based on niche difference, native species repel invasive plants through competitive exclusion (MacDougall, Gilbert, & Levine, 2009). A theory of limiting similarity originated from classical competition theories (Macarthur & Levins, 1967; Weltzin, Muth, Von Holle, & Cole, 2003), and the theory proposes that there is a limit to niche overlap or similarity in resource use between native species and invading species. Based on mechanisms of competition, invading species cannot establish in a niche similar to that of a native species (Funk et al., 2008). When niches overlap, the species with superior fitness (competitive ability) will prevail (MacDougall et al., 2009).

Second, the diversity effect is an indication of how combinations of species resist invasion. This effect is related to niche partitioning, which leads to coexistence among native species and the diversity effect on invasive plants. According to the diversity–resistance hypothesis (Elton, 1958), the uptake of available resources and the occupation of niches are more complete in a species‐rich community, which prevents invasion. Niche partitioning is observed when resources are partitioned as the species of neighboring plants and the canopy complexity increase (Ashton, Miller, Bowman, & Suding, 2010; Booth, Caldwell, & Stark, 2003; Frankow‐Lindberg, 2012). Summarizing the research to date, compared with one particular alternative species, developing a seed mixture that contains three or four species will lead to a diverse plant community that can maintain biotic resistance in a changing environment and prevent re‐invasion by invasive plants.

Functional traits are defined as morpho‐physio‐phenological traits (Cornelissen, 2003; Violle et al., 2007) that are linked with the niche and fitness of a species (Drenovsky et al., 2012; Eisenhauer, Schulz, Scheu, & Jousset, 2013; Funk et al., 2008). Functional groups are group of species whose traits are similar to each other. According to Fox's assembly rule, when a native community lacks a particular functional group, the community is easily invaded by an invader that belongs to that functional group based on limiting similarity (Fox, 1987; Von Holle & Simberloff, 2004). In several studies, a predefined functional group, such as one defined by growth form, was used to test the effect of functional groups on invasion (Booth et al., 2003; Gooden & French, 2015; Pokorny et al., 2005; Prieur‐Richard, Lavorel, Grigulis, & Dos Santos, 2000; Sheley & James, 2010; Symstad, 2000; Tilman, 1997b; Von Holle & Simberloff, 2004), but these particular groups often ignored functional traits that might be relevant to biotic resistance. For example, some invasive species exploit temporal niches when these are not occupied by other species (Wilsey, Daneshgar, & Polley, 2011; Wolkovich & Cleland, 2010); therefore, functional traits related to life‐history strategies, such as life span, can determine the timing of species establishment and possible competitive interactions. Species that establish early and grow rapidly may pre‐empt niches, leading to inhibition of the slow‐growing species in a community assemblage (Mwangi et al., 2007). Therefore, the classification of species into functional groups based on several relevant traits is essential to relate functional group identity with biotic resistance.

Diversity–interaction models (Kirwan et al., 2009) permit predictions of the relationship between diversity and biotic resistance across communities of different compositions by comparing different models based on different ecological assumptions concerning species interactions. When combined with a functional group approach, diversity–interaction models promise to reveal new insights into mechanisms of resistance to invasion (Frankow‐Lindberg, 2012; Frankow‐Lindberg, Brophy, Collins, & Connolly, 2009).

The focus of this research was on the processes that influence the outcome of community assembly when a site is disturbed, with the goal to limit establishment of an invasive species such as *A. altissima*. Successful establishment of *A. altissima* depends on the level of biotic resistance, which is the ability of other plant species to limit the success of invasions. Therefore, the primary objective was to understand the determinants of biotic resistance to invasion in a plant community assembly using *A. altissima* as a model. Based on the assumption that some species, or combination of species, are more resistant to invasion than others, we hypothesized that certain functional groups will be most resistant to invasion, whereas the species effect will be redundant within each functional group. Based on the hypothesis of limiting similarity, we hypothesized that the functional group of which *A. altissima* was a member would be more resistant than other groups. Additionally, we hypothesized that a mixture of species will be more resistant to invasion than a monoculture of a species (a diversity effect), and we asked how species interact to produce such a diversity effect.

## 2 Materials and Methods

### 2.1 Species selection and functional classification

Twenty‐two species were selected based on expert opinion of the invaded system and availability of seed. To address the hypotheses, existing functional trait information for the study species was sourced. The TRY trait database (Kattge et al., 2011) was used to obtain the functional traits of species, and specific leaf area, canopy height, life span, growth form, woodiness, relative growth rate, and leaf dry matter content were selected. These functional traits are relevant to the list of common core plant traits related to dispersal, establishment, and persistence (Weiher et al., 1999) and were also related to competitive ability and growth (Funk et al., 2008). To build a species‐trait matrix, the median value of a measured trait per species was used for data to be consistent. Species were classified into functional groups based on trait similarity. Based on these functional traits, Gower's similarity coefficient among species was calculated using the *gowdis* function in the R statistical software package (Gower, 1971; Podani, 1999). All traits were standardized and equally weighted in the calculation of the similarity coefficient.

**Figure 1:**
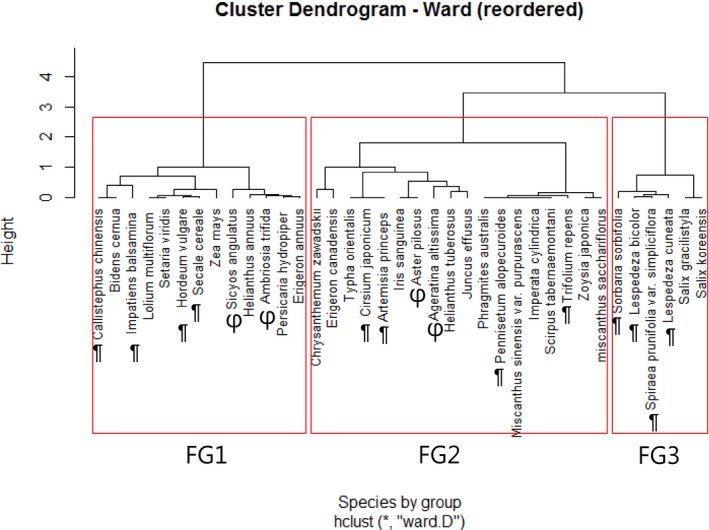
Functional classification of species. “¶” refers to testing alternative resident plants in the experiment. “φ” refers to major invasive plants in the area of the capital Seoul, South Korea. Among the invasives, the target invasive plant in this experiment was *Ageratina altissima*

Including the 22 species, 37 total plant species for broad interpretation, which included four typical invasive plants (*Ambrosia trifida*,* Ageratina altissima*,* Sicyos angulatus*, and *Aster pilosus*), in the capital area of Seoul, South Korea, were classified into three functional groups with the cluster analysis with ward option using the *hclust* function in the R program (Figure 1). Functional groups differed from one another primarily by life span, growth form, and woodiness traits. The three functional groups were FG1 for annual plants, FG2 for perennial herbaceous plants, and FG3 for perennial woody plants. Details of the characteristics of each functional group are shown in Table 1. Species nomenclature and status (native or introduced) in this study followed the Flora of North America (Flora of North America Editorial, 1993) and the database of Vascular Plants of Canada (VASCAN), respectively.

**Table 1:** Functional group trait characteristics

Trait	FG1	FG2	FG3	Units
Life longevity	Annual	Perennial, biennial	Perennial	
Growth form	Herb, grass, forb	Herb, forb, sedge, grass	Shrub, tree	
Woodiness	Non‐woody	Non‐woody	Woody	
SLA	25.13 ± 4.10	26.95 ± 19.23	25.90 ± 11.12	m^2^/kg
RGR	0.22 ± 0.05	0.17 ± 0.12		g g^−1^ day^−1^
LDMC	3.57 ± 6.88	6.30 ± 10.31	8.23 ± 15.59	g/g
Height	140.3 ± 135.9	70.36 ± 55.16	156.5 ± 56.3	cm

### 2.2 Experimental setup and seed preparation

A pot experiment was set up in a greenhouse facility in the School of Biological Sciences at Seoul National University. The experiment was designed to simulate a situation in which seeds of *A. altissima* reach bare soil after a biological disturbance. Pots were 22 cm in diameter and 30 cm in height, and the soil used in the experiments was a fertile agricultural soil.

Seeds of *A. altissima* were collected on the campus of Seoul National University in November 2015. Most seeds of native plants were purchased from seed suppliers. Seed viability among native plants was standardized by applying the identical number of viable seeds per species to experimental units. To determine pure live seeds, a germination test was conducted. All seeds were cold‐stratified at 3°C before the germination test, following standard methods (Lindig‐Cisneros & Zedler, 2001). Before the experiment, 100 seeds per species were placed in each of three Petri dishes with filter paper (Whatman^®^ No. 1) moistened with 6 ml of distilled water under fluorescent light. The species with a germination rate below 3% were excluded. Among 22 species, the germination rate was above 3% for only 12 species. Viable seeds per species, not seedlings, were applied in the pot experiments.

### 2.3 Design of competition test

An additive competition design was applied to test the competitive effect of resident species on *A. altissima* (Connolly, Wayne, & Bazzaz, 2001; Keddy, Twolan‐Strutt, & Wisheu, 1994; Snaydon, 1991). Each treatment pot received the seeds of *A. altissima* and those of native plants. For the 12 monoculture treatments, one native species per pot was used. For the seven mixture treatments, four randomly chosen native species per pot were used. Control pots received only seeds of *A. altissima*. All species in monocultures or mixtures were sown in early March 2016 with the seeds of *A. altissima* in treatments or the control. Each seeding density of native plant(s) and *A. altissima* was total 300 viable seeds per each pot. Control pot received 300 viable seeds of *A. altissima* only. The sowing density was approximately 8,000 live seeds/m^2^. Treatments were applied in a randomized complete block design, with three replicates per treatment.

### 2.4 Data measurement and analyses

At the end of July 2016, the number of shoots, aboveground biomass, plant height, and plant cover of *A. altissima* in each treatment and control pot were measured to calculate the primary response variable (see below). Additionally, plant cover, plant height, and aboveground biomass of all native plants were measured to correlate these variables with the response variables. For aboveground biomass, the aboveground portion of plants was harvested at the end of July and then weighed following drying at 80°C for 48 hr. Plant height was estimated for each species to the closest 0.5 cm. The RCI (relative competition index) was calculated to estimate the competitive effect of native plant(s) on *A. altissima* using the following equation (Weigelt & Jolliffe, 2003):

(1) $${\text{RCI}}_{Y}=\frac{Y_{\mathrm{control}}-Y_{\mathrm{treatment}}}{Y_{\mathrm{Control}}}$$

where RCI is the relative competition index of a native plant on *A. altissima* in either monoculture or mixture for a given variable Y (i.e., number of shoots, aboveground biomass, plant height, or plant cover of *A. altissima*). Y_control_ is the performance of *A. altissima* in the control, and Y_treatment_ is the performance of *A. altissima* in a treatment. Because RCI_number of shoots_, RCI_biomass_, RCI_height_, and RCI_plant cover_ were highly correlated with one another, RCI_avg_ was calculated, which is the arithmetic mean of RCI_number of shoots_, RCI_biomass_, RCI_height_, and RCI_plant cover_, as the primary response variable for all analyses. A value of 0 for RCI_avg_ indicated no competitive effect on *A. altissima*, a value of 1 indicated complete competitive exclusion of *A. altissima*, and a negative RCI indicated facilitation of the establishment and growth of *A. altissima* by native plants.

For the monoculture treatments in the experiment, ANOVA was used to test for functional group identity effect and species identity effect nested within each functional group on RCI_avg_. A generalized linear mixed model (REML; *F*‐test) was used for this test to account for the random block effect (Bolker et al., 2009). Normality of residuals and homoscedasticity were evaluated, and the response variables were transformed when necessary. When a significant functional group effect was detected, the means of functional groups were compared using a contrast test on each pair of functional groups. When a significant species identity effect within each functional group was detected, Tukey's HSD multiple comparison test was used to compare means of species identity effect for each functional group.

### 2.5 Diversity–interaction models

Diversity–interaction models (Kirwan et al., 2009) were used to investigate species interaction patterns that contributed to biotic resistance in the mixture treatments. Comparisons of models based on different ecological assumptions were used to test alternative hypotheses about the relative role of functional groups and functional redundancy in biotic resistance (Kirwan et al., 2009).

Model 1 describes the species identity effect alone without species interaction:

(2) $$y=\sum_{i=1}^{s}\beta_{i}P_{i}+\varepsilon$$

The response variable (y) represents RCI_avg_ as an indicator of biotic resistance to invasion by *A. altissima*. β_*i*_ is the estimated performance of species *i* as a contribution to biotic resistance, and *P*_*i*_ is the initial proportion of species *i* in a seed mixture. For monoculture treatments of species *i*,* P*_*i*_ is equal to 1.

Model 2 describes the functional group identity effect alone without species interaction:

(3) $$y=\beta_{\mathrm{FG}1}P_{\mathrm{FG}1}+\beta_{\mathrm{FG}2}P_{\mathrm{FG}2}+\beta_{\mathrm{FG}3}P_{\mathrm{FG}3}+\varepsilon$$

where β_FG1_ is the estimated functional group identity effect of FG1 and *P*_FG1_ is the sum of all species proportions within that FG1.

Model 3 describes the functional group identity effect and average species interaction:

(4) y=βFG1PFG1+βFG2PFG2+βFG3PFG3+δav∑i,j=1i<jsPiPj+ε

where δ_av_ is the single interaction coefficient assuming that a pair of species interacts equally to contribute to such a diversity effect.

Model 4 describes the functional group identity effect and species interactions within and between functional groups:

(5) y=βFG1PFG1+βFG2PFG2+βFG3PFG3+δwFG1∑i,j=1i<jtPiPj+δwFG2∑i,j=t+1i<jt+hPiPj+δwFG3∑i,j=t+h+1i<jsPiPj+δbFG1·FG2PFG1PFG2+δbFG1·FG3PFG1PFG3+δbFG2FG3PFG2PFG3+ε

where δ_wFG1_ is the coefficient of pairwise species interaction within FG1 and δ_bFG1FG2_ is the coefficient of pairwise species interactions between FG1 and FG2.

Model 5 describes the functional group identity effect and separate pairwise species interactions:

(6) y=βFG1PFG1+βFG2PFG2+βFG3PFG3+βFG4PFG4+∑i,j=1i<jsδijPiPj+ε

where δ_*ij*_ is the coefficient of separate pairwise interaction between species *i* and species *j*.

Model 6 describes the functional group identity effect and species interactions between functional groups without species interactions within each functional group:

(7) $$\begin{array}{cc} y= & \beta_{\mathrm{FG}1}P_{\mathrm{FG}1}+\beta_{\mathrm{FG}2}P_{\mathrm{FG}2}+\beta_{\mathrm{FG}3}P_{\mathrm{FG}3}+\delta_{\mathrm{bFG}1\cdot \mathrm{FG}2}P_{\mathrm{FG}1}P_{\mathrm{FG}2}\\ & +\delta_{\mathrm{bFG}1\cdot \mathrm{FG}3}P_{\mathrm{FG}1}P_{\mathrm{FG}3}+\delta_{\mathrm{bFG}2\cdot \mathrm{FG}3}P_{\mathrm{FG}2}P_{\mathrm{FG}3}+\varepsilon \end{array}$$

Each model was tested using the glm function in the R statistical software package. Pairs of models were compared for a significant difference in model predictions for RCI_avg_ using the ANOVA function in the R software. Akaike's information criterion (AIC) was used to compare and select models (Burnham, Anderson, & Burnham, 2002).

All ANOVA tests and correlation analyses were performed using JMP software (© SAS Institute Inc., Cary, NC, USA). Cluster analysis and diversity–interaction modeling, which are based on multiple regressions, were conducted using the R program (R Development Core Team, 2015).

Data are available from the Figshare Digital Repository: https://dx.doi.org/10.6084/m9.figshare.3593049.v1.

## 3 Results

### 3.1 Monoculture treatments

**Figure 2:**
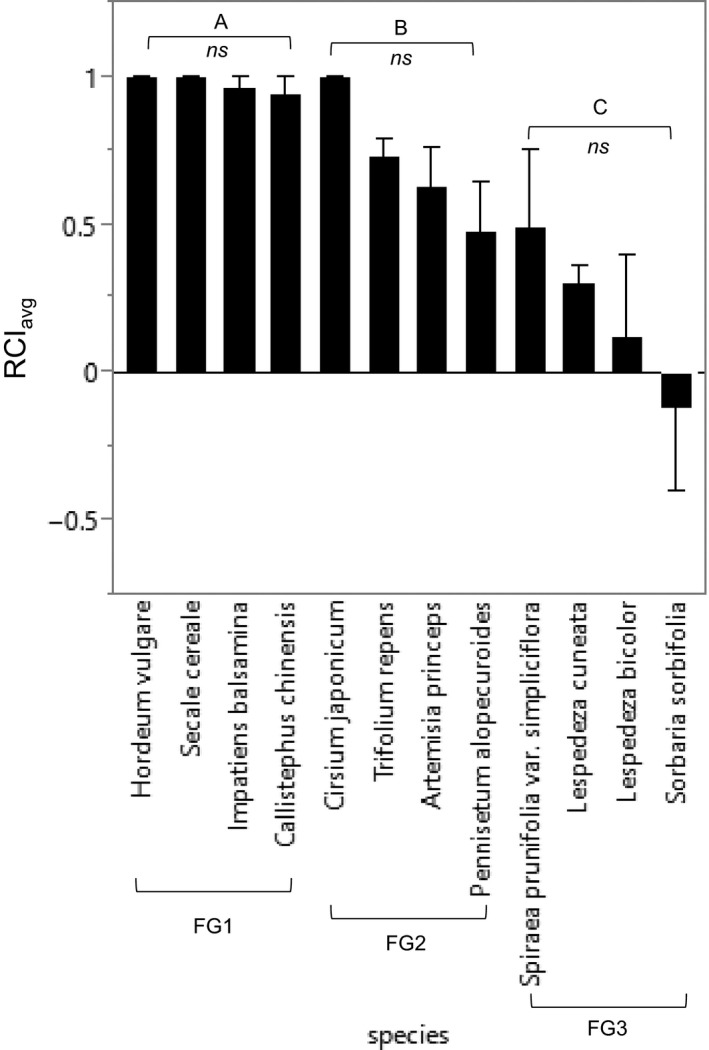
Results of the monoculture treatments. RCI_avg_: relative competition index of resident plant(s) as an indicator of biotic resistance (see Equation (1)). Each species was grouped by functional group 1, 2, or 3. The same letter indicates that means are not significantly different from one another (functional group). *ns* indicates no significant difference among species within each functional group. Error bar represents the standard error

In monoculture treatments, the relative competitive effect of resident plants on *A. altissima* was primarily related to their functional group identity, whereas the species identity effect remained redundant within each functional group (Figure 2). The relative competitive index (RCI_avg_) of the 12 resident plants on *A. altissima* was significantly different among the three FGs (*F*_2,31_ = 22.84, *p *<* *.001), but was not significantly different within each FG (FG1: *F*_3,6_ = 1.00, *p *=* *.4547; FG2: *F*_3,6_ = 4.07, *p *=* *.067; and FG3: *F*_3,6_ = 1.17, *p *=* *.3937). The highest RCI_avg_ was for FG1 (annual plants), followed by FG2 and FG3 (mean RCI_avg_ = 0.975, 0.710, and 0.196, respectively; Figure 2).

**Figure 3:**
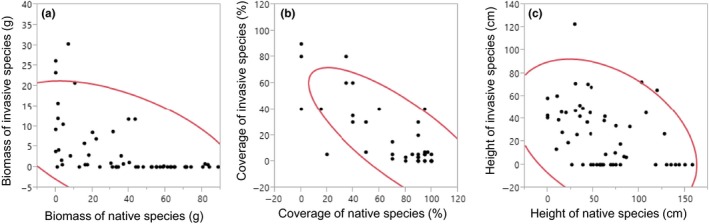
Relationship between native plants and the invasive plant, *Ageratina altissima*, based on (a) biomass, (b) coverage, and (c) height. Correlations were significant for all three measures (Pearson correlation coefficients were −0.536, −0.792, and −0.383, respectively)

The performance traits of resident plants were significantly negatively correlated with the biomass of *A. altissima* (Pearson coefficients: *r *=* *−0.536), plant cover (*r *=* *−0.792), and height (*r *=* *−0.383; Figure 3). Among the plant functional traits used to classify functional group, relative growth rate (*r *=* *0.923), seed and LDMC (*r *=* *−0.5535) were significantly correlated with RCI_avg_, and annual plants with grass and herb in growth form and non‐woody plant species showed relatively high RCI_avg_ (Appendix S1).

### 3.2 Mixture treatments

**Figure 4:**
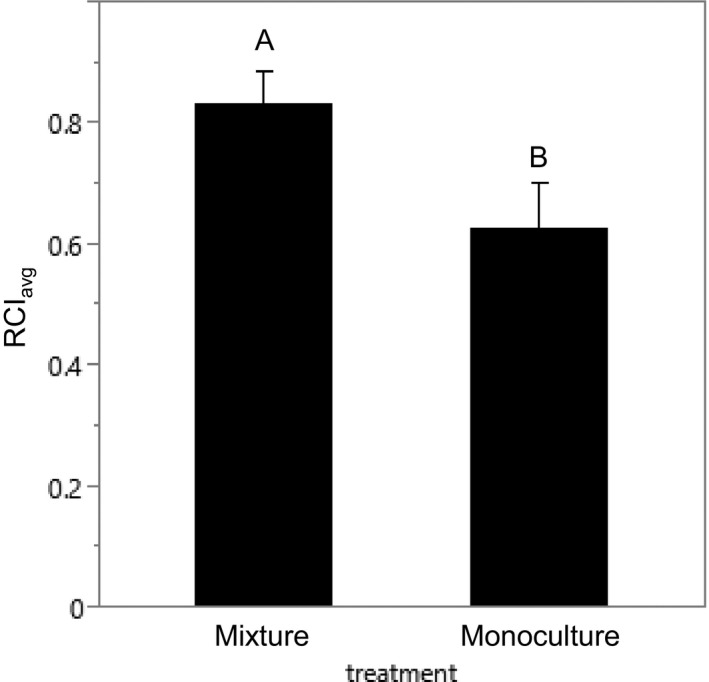
Comparisons between monoculture and mixture treatments. RCI_avg_: relative competition index of resident plant(s) as an indicator of biotic resistance (see Equation (1)). The same letter indicates that means are not significantly different from one another. Error bar represents the standard error

Mixtures of resident plants were more resistant to invasion than monocultures, and this diversity effect on biotic resistance was the result of positive interactions between FG1 and FG3 (Figuress 4 and 5). RCI_avg_ was significantly greater in mixtures than in monocultures (*F*_1,53_ = 4.08; *p *=* *.048; Figure 4). Aboveground biomass of resident species was also significantly greater in mixture treatments than in monoculture treatments (*F*_1,53_ = 7.33; *p *<* *.009).

**Figure 5:**
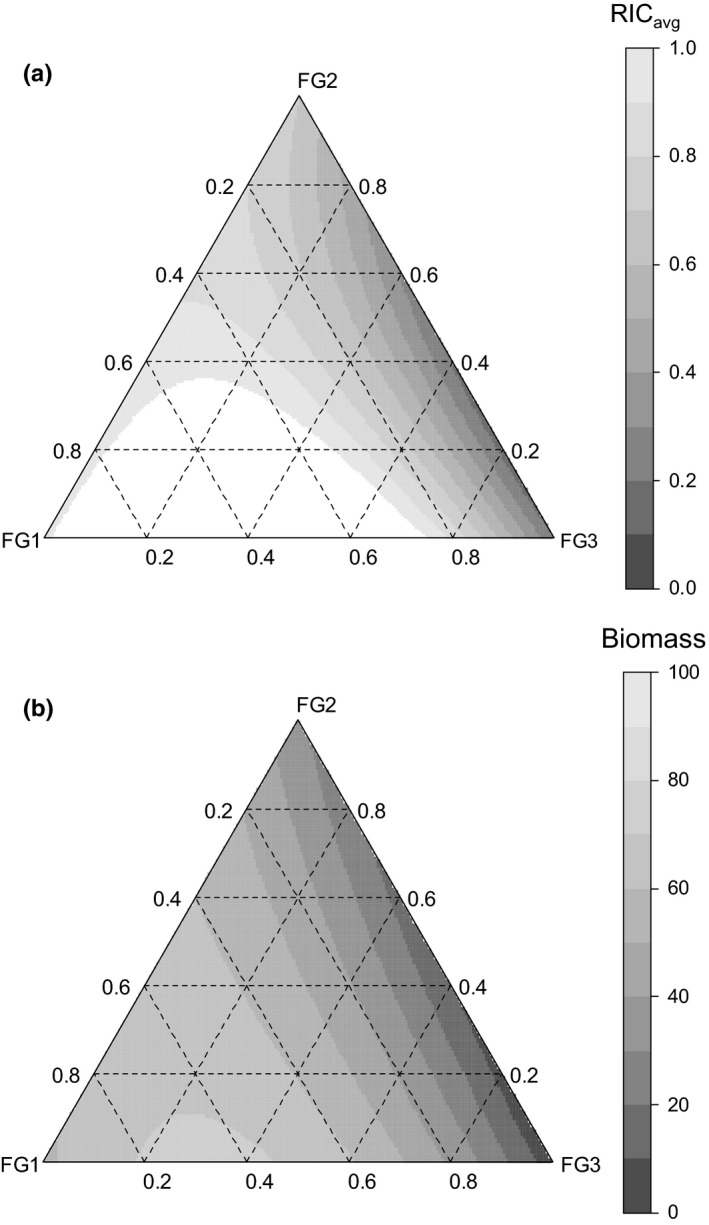
Predictions of biodiversity–interaction models on the effect of functional group composition on (a) RCI_avg_: relative competition index of resident plant(s) as an indicator of biotic resistance (see Equation (1)) and (b) aboveground biomass (g) of resident plant(s) per pot. Each corner of the ternary plot represents the monoculture of each functional group, whereas the inner area of the plot represents the mixtures of functional groups. For details on the model equation, see Equation (7)

Comparisons between pairs of diversity–interaction models fitted to the experimental data set revealed distinctive species interaction patterns by functional group that contributed to biotic resistance. The functional group identity effect terms fitted as well as species identity effect terms (Model 1 vs. Model 2; *F*‐test; *p *=* *.171, AIC: 30.99 vs. 27.26). The evidence was strong for an average interaction term effect (diversity effect) (Model 2 vs. Model 3; *F*‐test; *p *=* *.003, AIC: 27.26 vs. 20.82). The species interaction by functional group (species interaction within and between functional groups) terms fitted much better than the single average interaction term (Model 3 vs. Model 4, *F*‐test; *p *=* *.001, AIC: 20.82 vs. 11.55). However, the separate pairwise species interactions terms did not fit better than the species interaction by functional group term (Model 4 vs. Model 5; *F*‐test; *p *=* *.579; AIC: 11.55 vs. 13.18). No evidence of significant species interaction within each functional group was detected (Model 4 vs. Model 6, *F*‐test; *p *=* *.725, AIC: 11.55 vs. 7.09). Thus, Model 6 (functional group identity effect and species interaction between functional groups) was chosen for the final model prediction because it fitted as well as the complex models with separate pairwise species interactions. Figure 5a shows Model 6 predictions on the effect of functional group composition in seed mixtures on biotic resistance to invasion by *A. altissima*. Figure 5b shows the identical model prediction when the model was fitted to aboveground biomass instead of RCI_avg_ of resident plants. In either case, a positive interaction was found between FG1 and FG3 in their contribution to biotic resistance. The highest RCI_avg_ and aboveground biomass were estimated for a mixture of FG1 and FG3 at a ratio of 2 to 1, approximately.

## 4 Discussion

### 4.1 Functional groups and biotic resistance

In this study, functional group identity determined biotic resistance to invasion by *A. altissima*, but the species identity effect was redundant within each functional group. The most resistant functional group was FG1 (fast‐growing annuals), which was a different functional group from *A. altissima* (FG2). Thus, this result did not support the role of limiting similarity in biotic resistance. However, a significant role of functional groups in biotic resistance was found, with some exceptions (Von Holle & Simberloff, 2004), in other studies that tested functional groups based on various plant traits such as life longevity, growth form, root structure, plant height, or phytosynthetic pathways (Byun et al., 2013; Prieur‐Richard, Lavorel, Dos Santos, & Grigulis, 2002b; Prieur‐Richard, Lavorel, Linhart, & Dos Santos, 2002a; Sheley & James, 2010; Wang, Ge, Zhang, Bai, & Du, 2013). The functional group that most resisted invasion was not always consistent among those studies. In some cases, the functional group most similar to the invasive plant offers the most resistance (Bakker & Wilson, 2004; Dukes, 2002; Fargione, Brown, & Tilman, 2003; Hooper & Dukes, 2010; Mwangi et al., 2007; Pokorny et al., 2005; Turnbull et al., 2005), indicating limiting similarity, whereas, in other cases, different functional groups offer the most resistance to invasion (Byun et al., 2013; Lulow, 2006; Sheley & James, 2010), suggesting fitness inequality as one of the key mechanisms of resistance. Invasion success may depend on both fitness and niche differences with resident species (MacDougall et al., 2009). We demonstrated the important role of a pre‐emptive effect (first come, first served) in the control of invasive plants which is consistent with another study (Stuble & Souza, 2016), and early emergence increased components of plant fitness, such as seedling growth, in a controlled experiment (Verd & Traveset, 2005).

In the present study, biomass, coverage, height, and relative growth rate were important to control the invasion by *A. altissima*. The indicators of fitness and biotic resistance are plant height (Gaudet & Keddy, 1988), biomass (Gaudet & Keddy, 1988; Lulow, 2006; Rinella, Pokorny, & Rekaya, 2007), plant cover (Bakker & Wilson, 2004; Gerhardt & Collinge, 2003), and plant size (Schamp & Aarssen, 2010). In particular, biomass is an indicator of plant competitive ability (Gaudet & Keddy, 1988) and biotic resistance (Lulow, 2006; Rinella et al., 2007).

### 4.2 Diversity effect on biotic resistance

In this study, we observed a diversity–resistance relationship, which is consistent with previous community‐scale experimental studies on multiple invaders (Abernathy, Graham, Sherrard, & Smith, 2015; Byun et al., 2013; Frankow‐Lindberg et al., 2009; Henriksson, Yu, Wardle, Trygg, & Englund, 2016; Stachowicz & Byrnes, 2006), but see also other studies (Henriksson, Yu, Wardle, & Englund, 2015; Schamp & Aarssen, 2010). Similar patterns at a community scale are reported from field observations (Brown & Peet, 2003; Levine, 2000), but opposite patterns are observed at larger scales (Brown & Peet, 2003; Levine, 2000; Stohlgren, Barnett, & Kartesz, 2003; Stohlgren et al., 1999). Spatially covarying environmental factors such as resource availability can affect both diversity and invasibility (Byers & Noonburg, 2003; Davies, Harrison, Safford, & Viers, 2007a; Levine & D'Antonio, 1999). Furthermore, different ecological processes such as dispersal and species recruitment can predominate at a larger scale (Fridley et al., 2007; Pauchard & Shea, 2006; Tilman, 1997a). Notably, the diversity effect was best described by positive species interactions between functional groups in this study, which implied niche partitioning among species in a mixture. This diversity effect implies complementarity rather than selection effects for the control of invasions by biotic resistance (Loreau, 1998; Loreau & Hector, 2001). Functionally diverse resident communities use resources more completely than a simple community (Davies, Pokorny, Sheley, & James, 2007b; Pokorny et al., 2005; Prieur‐Richard et al., 2000; Rinella et al., 2007). Furthermore, in functionally diverse communities with a complex canopy, less light penetrates through the canopy (Frankow‐Lindberg, 2012; Lindig‐Cisneros & Zedler, 2002).

### 4.3 Case studies to restore resistant plants to control invasion

Several restoration experiments have been conducted to test biotic resistance in an invasion context. Unfortunately, a case study for the control *A. altissima* has not yet been conducted. Based on this study, restoration of native plant cover controlled up to 100% of *A. altissima* establishment. *Phragmites australis* is an exotic invasive plant in the wetlands of North America, and, in an experiment in a salt marsh, the transport of a halophyte into the marsh reduced rhizome growth of *Phragmites australis* by 60% (Peter & Burdick, 2010). A diversity effect (mixtures are more resistant than monocultures of a species) was also found with the selection effect of *Spartina alterniflora*, and, in a freshwater mesocosm experiment, biotic resistance was significantly different among functional groups (Byun et al., 2013). In the present study, the strongest biotic resistance was found in rapidly growing annual plants that showed a priority effect. In another experiment that examined environmental effects, flooding always reduced invasion success, and flooding assisted or inhibited biotic resistance depending on the adaption of species to the environment (Byun et al., 2015). Biotic resistance is particularly effective with low propagule pressure (Byun et al., 2015). Other studies also examined the restoration of native plants to control invasive plants, including *Centaurea diffusa* (Meiman, Redente, & Paschke, 2009), *Centaurea solstitialis* (Dukes, 2001, 2002), *Rapistrum rugosum* (Cutting & Hough‐Goldstein, 2013; Simmons, 2005), *Cardaria draba*,* Cirsium arvense*,* Bromus tectorum* and *B. japonicas* (Perry, Cronin, & Paschke, 2009), *Arundo donax* (Quinn & Holt, 2009), *Agropyron cristatum* (Bakker & Wilson, 2004), and *Persicaria perfoliata* (Cutting & Hough‐Goldstein, 2013).

Based on empirical evidence, restoring plant species increases biotic resistance. Depending on seed configuration, environment, and invasive plant, plant restoration results in an approximate reduction in invasion ability of 50–100%. In most experiments, invasive plants survived regardless of plant restoration, with the exception of those in the present experiment. In conclusion, biotic resistance alone may not prevent invasive plants completely (Levine, Adler, & Yelenik, 2004); however, biotic resistance contributes to constraining the abundance of invasive plants and determines the identity of an invasive plant at regional scales (Davies, Cavender‐Bares, & Deacon, 2011; Fargione et al., 2003).

### 4.4 Implications for management

Restoration has many advantages over methods of eradication; restoration is self‐regenerative (not requiring repeated application), is less of a threat to native, desirable species, and prevents disturbance that stimulates re‐invasion by invasive plants (Simmons, 2005). Therefore, restoration of native plant cover is an alternative, innovative method to protect native species from invasive plants. Practical designs for restoration include some technical questions that must be answered such as how to select and how to combine species for restoration and how to identify appropriate environmental conditions for restoration. However, information on the use of native plant restoration as an alternative to control invasions continues to be lacking (Hazelton, Mozdzer, Burdick, Kettenring, & Whigham, 2014).

*Ageratina altissima* is a noxious weed and an invasive plant that is very difficult to control (Chun et al., 2001; Kim, Jang, & Park, 2014; Lee, Yoo, & Lee, 2003; Lee et al., 2005). In the field, invasion success is determined by the interplay among environmental conditions, propagule pressure, and biotic resistance (Catford, Jansson, & Nilsson, 2009; D'Antonio, 1993; Dethier & Hacker, 2005; Perelman, Chaneton, Batista, Burkart, & León, 2007). Although this experiment did not test all these factors affecting invasion, the approach generally revealed the importance of biotic resistance. The forest edge, disturbed by human activities such as trampling and creation of paths, is the site for much of the invasion by this invasive plant (Chun et al., 2001; Kim et al., 2014; Lee et al., 2003, 2005), and *A. altissima* was identified as an indicator species of edge effect following recent silvicultural clearcutting in a mixed mesophytic forest (Landenberger & Ostergren, 2002). Trampling has a role in the invasion window of *A. altissima*, and sites in which they grow vigorously show low species diversity due to their dense cover (Lee et al., 2003). Human activities that result in disturbance increase the susceptibility of most ecosystems, which highlights the requirement, when applicable, to minimize damage to the matrix of vegetation cover and/or facilitate the rapid establishment of competitive cover with the goal to restore disturbed habitat. In these cases, the results of this study showed that functional group identity and diversity of resident or restored plant communities were good indicators of potential biotic resistance to seed‐mediated invasion by *A. altissima*. We suggest the use of FG1 species, such as *Hordeum vulgare* and *Secale cereal*, primarily, to restore native plant cover to suppress invasion by *A. altissima* and also mixing FG1 with FG3 species for niche partitioning and potential long‐term effects. Concerning propagule pressure, most field situations are expected to have much lower seed pressure than the level tested in this experiment; however, even then, complete competitive exclusion may not be achieved. Follow‐up monitoring and selective control of *A. altissima* establishment could be necessary. The distribution of *A. altissima* is closely correlated with the soil contents of total nitrogen and available phosphorus (Suh, Kil, Kim, & Lee, 1997), and *A. altissima* is adaptable to a broad range of soil conditions (Kim et al., 2014); therefore, controlling for these elements will help to further suppress the growth of *A. altissima*. Growth of *A. altissima* is highest at a light intensity of 7,500 lux (Suh et al., 1997) and is correlated with decreasing litter depth (Kim et al., 2014); thus, increased cover of native plants will decrease light intensity and increase litter depth to contribute to the suppression of this plant.

The present study indicates that the guiding ecological principles to understand and/or manage, if desired, biological invasions could emerge from advances in community theory and the use of a functional framework. To facilitate generalization, widely distributed invasive plants should be targeted in different contexts and the results should be scaled‐up to field conditions.

## Authors' Contribution

CB and EL conceived the research. CB designed the research, performed the experiment, analyzed data, and wrote the manuscript. CB and EL edited the manuscript. All authors contributed critically to the drafts and gave final approval for publication.

## Conflict of Interest

None declared.
