# Supplementary material for: Ecological application of biotic resistance to control the invasion of an invasive plant, Ageratina altissima
Source: Ecol Evol. 2017 Mar 2;7(7):2181–92. doi: 10.1002/ece3.2799 (PMC5383480; doi:10.1002/ece3.2799)
Supplement: Supplementary file 1 [file ECE3-7-2181-s001.docx]

**Appendices**

**Appendix S1**. Relationship between functional traits and biotic resistance (RCI_avg_) in the experiment. Functional traits (from TRY trait database): (a) life longevity, (b) growth form, (c) woodness, (d) height, (e) SLA, (f) RGR, and (g) LDMC. Solid line represents linear regression fit. Means connected by same letter are not significantly different from each other in ANOVA test (life longevity: *F*_1,34_=17.14; *P*<0.001; growth form: *F*_2,30_=14.80; *P*<0.001; woodiness: *F*_1,34_=35.99; *P*<0.001). †*r* values represent Pearson correlation coefficient. ‡*P* values represent *t* test result on slope in linear regression analysis.
